# Multi-therapeutic strategy targeting parasite and inflammation-related alterations to improve prognosis of chronic Chagas cardiomyopathy: a hypothesis-based approach^*^

**DOI:** 10.1590/0074-02760220019

**Published:** 2022-03-23

**Authors:** Joseli Lannes-Vieira

**Affiliations:** 1 Fundação Oswaldo Cruz-Fiocruz, Instituto Oswaldo Cruz, Laboratório de Biologia das Interações, Rio de Janeiro, RJ, Brasil

**Keywords:** Chagas disease, chronic chagasic cardiomyopathy, preclinical tests, benznidazole, pentoxifylline, multi-therapy

## Abstract

Chagas disease (CD), caused by infection by the protozoan parasite *Trypanosoma cruzi*, presents as main clinical manifestation the chronic chagasic cardiomyopathy (CCC). CCC afflicts millions of people, mostly in Latin America, and vaccine and effective therapy are still lacking. Comprehension of the host/parasite interplay in the chronic phase of *T. cruzi* infection may unveil targets for rational trait-based therapies to improve CCC prognosis. In the present viewpoint, I critically summarise a collection of data, obtained by our network of collaborators and other groups on CCC and preclinical studies on pathogenesis, targeting identification for intervention and the use of drugs with immunomodulatory properties to improve CCC. In the last two decades, models combining mouse lineages and *T. cruzi* strains allowed replication of crucial clinical, histopathological, and immunological traits of CCC. This condition includes conduction changes (heart rate changes, arrhythmias, atrioventricular blocks, prolongation of the QRS complex and PR and corrected QT intervals), ventricular dysfunction and heart failure, CD8-enriched myocarditis, tissue remodeling and progressive fibrosis, and systemic inflammatory profile, resembling “cytokine storm”. Studies on Chagas’ heart disease pathogenesis begins to unveil the molecular mechanisms underpinning the inflammation-related cardiac tissue damage, placing IFNγ, TNF and NFκB signaling as upstream regulators of miRNAs and mRNAs associated with critical biological pathways as cell migration, inflammation, tissue remodeling and fibrosis, and mitochondrial dysfunction. Further, data on preclinical trials using hypothesis-based tools, targeting parasite and inflammation-related alterations, opened paths for multi-therapeutic approaches in CCC. Despite the long path taken using experimental CD models replicating relevant aspects of CCC and testing new therapies and therapeutic schemes, these findings may get lost in translation, as conceptual and economical challenges, underpinning the valley of death across preclinical and clinical trials. It is hoped that such difficulties will be overcome in the near future.

Chronic chagasic cardiomyopathy (CCC): a hallmark of Chagas disease

Inflammation-related tissue injury associated with low-grade parasite persistence, progressive fibrosis, cardiac dysfunction, potentially evolving to heart failure, and systemic inflammatory profile are hallmarks of chronic Chagas’ heart disease. This is the main clinical manifestation of Chagas disease (CD), a neglected tropical disease caused by the protozoan parasite *Trypanosoma cruzi*, discovered more than hundred and twenty years ago. CD afflicts 7-8 million people, mostly in endemic areas of Latin America, and human migration contributes to globalisation of this health problem.^1^ Prophylactic and therapeutic vaccines are not available.^2^ Currently, most chronically CD patients with access to clinical follow-up are only prescribed with signs- and symptoms-based treatments.^1^ ^,^ ^3^

After *T. cruzi* infection, acute phase lasts four to eight weeks. In this phase, the parasite is detected in circulating blood and tissues, triggering an inflammation-related immune response.^4^ The immune response generated in the acute phase, and still present in the chronic phase, with the production of antibodies and activation of effector T-cells, may control the intense parasitism and parasitaemia, which becomes low and intermittent, however it fails to eliminate the parasite.^4^ The molecular mechanisms contributing to the persistence of *T. cruzi* parasite amastigote forms in tissues and, particularly, inside certain cells as cardiomyocytes, in the chronic phase of CD need to be identified. In the acute and chronic phases, parasite antigens and extracellular vesicles containing *T. cruzi* molecules and cargos of parasite-host cell interactions are detected in plasma and tissues. In this way, low-grade tissue parasitism may contribute to continuous stimulation of tissue cells and, particularly, of cells of the immune system.^5^ In the chronic phase, high titers of antibodies (IgG) are biomarker of diagnosis and disclose persistence of the infection, being seroconversion a potent, but time-consuming, cure criterion.^1^ Thus, therapies targeting parasite are a demand to add advantageous to immune-mediated parasite control and impact disease progression. Effectiveness of the offered etiological treatments targeting *T. cruzi*, benznidazole (Bz) and nifurtimox, is considered high in the acute phase of infection (60-80%), however most of the patients miss diagnosis and treatment. Pitifully, efficacy of these medicaments drops in the chronic phase of the infection (20-60%), possibly due to age of patients and differences in susceptibility to drugs among the different *T. cruzi* lineages.^1^ ^,^ ^6^ Randomised clinical trials based on parasite control in chronic CD patients aiming at interfering in disease progression have been used with controversial results.^6^ ^,^ ^7^ However, side effects remain as a shadow to use Bz in chronic CD patients. Thus, clinical trials (TRAENA, CHAGASAZOL, TESEO, BENDITA) are being conducted to reduce dose and/or time of administration of Bz, aiming at increasing adhesion to etiological therapy. New drugs accessing parasite metabolism and parasite-restrict biological pathways remain challenges to be faced.^6^

Decades after infection, most of the CD patients (70-80%) remain in the indeterminate form of CD, without clinical signs or symptoms. However, disease progresses in 20-30% of the patients (rate of 1.85-7% per year) to the symptomatic forms digestive, cardio-digestive and, mainly, cardiac form. The low-grade heart tissue parasitism and inflammation present in indeterminate patients may contribute to disease progression to cardiac form, with high morbidity and mortality, and impacting the public health system and family income, increasing poverty.^1^ ^,^ ^8^ Chronic chagasic cardiomyopathy (CCC), like other heart diseases, is clinically expressed by conduction changes, such as heart rate changes, arrhythmias, atrioventricular blocks, prolongation of the QRS complex and PR and corrected QT (QTc) intervals, with the possibility of progression to severe ventricular dysfunction and heart failure.^3^ The presence of mononuclear inflammatory cells infiltrating the heart tissue and the high rate of mortality are distinct features of CCC compared to other chronic myocardiopathies.^8^ Also, a systemic inflammatory profile, resembling a “cytokine storm” with increased serum concentrations of inflammatory (IL-6, TNF, IL-17, IFNγ) and regulatory (IL-4, IL-10, TGFβ) cytokines, and inflammatory mediators as nitric oxide (NO), is associated with the severity of Chagas’ heart disease but absent in non-cardiopathic and indeterminate form CD patients.^8^ ^,^ ^9^ ^,^ ^10^ ^,^ ^11^ Thus, in cardiopathic CD patients, low-grade parasite persistence coexists with inflammation-related tissue changes and a systemic inflammatory profile. Some years ago, we initiated our studies believing that the comprehension of host/parasite interplay contributing to CCC pathogenesis could unveil targets for rational trait-based modulatory therapies. Here, along with our network of collaborators and other groups’ work on CCC, I recapitulate and critically review a collection of data obtained, modelling proposals of multi-therapeutic strategies aiming at improving CCC prognosis.

Pathogenic mechanisms and modulatory preclinical strategy tests to CCC

Preclinical models of CD, combining mouse lineages and *T. cruzi* strains, emerge as tools to replicate parasitological, immunological and clinical features of CCC. The use of these models may allow (i) studies on molecular mechanisms underlying pathogenesis, (ii) unbiased screen studies, and (iii) hypothesis-based tests targeting potential pathogenic traits, thus contributing to identify therapeutic targets to be further tested in clinical trials aiming at improving CCC prognosis. Our results support that, according to the experimental model used, the initial host response to invader may define the severity of the chronic phase of the infection, therefore, opening an opportunity to test strategies to modify the clinical outcome, hampering progression or, preferentially, reversing CCC features. Different mouse lineages (C57BL/6 and C3H/He) infected with the same *T. cruzi* strain (Colombian, DTU TcI) show degrees of CCC severity (mild and severe), characterised by the intensity of electrocardiographic (arrhythmias, prolongation of PR and QTc intervals and QRS complex) and echocardiographic changes.^12^ ^,^ ^13^ In these models, the intensity of parasitaemia and heart parasitism in the acute phase of the infection was associated with the degree of CCC severity, manifested by the intensity of parasitism, inflammation, loss of the gap junction protein connexin-43 (Cx43) and progressive fibrosis in the heart tissue. In addition, CCC severity was directly related to CK-MB activity serum levels, a biomarker of cardiomyocyte injury, and systemic inflammatory profile, with increased serum levels of tumor necrosis factor (TNF) and nitric oxide (NO),^12^ ^,^ ^14^ ^,^ ^15^ thus replicating key aspects of chronic Chagas’ heart disease.^8^ ^,^ ^9^ ^,^ ^10^ ^,^ ^11^

In a proof-of-concept study to test TNF participation in CD pathogenesis, acutely *T. cruzi*-infected C3H/He mice were treated with the anti-TNF antibody infliximab. Survival was paralleled by reduced heart inflammation and tissue damage, without interference in parasite control,^16^ placing TNF as a key cytokine in the pathogenesis of *T. cruzi*-elicited cardiac disease. Moreover, all the results obtained after administration of the anti-TNF blocking antibody (infliximab) to chronically *T. cruzi*-infected C57BL/6 mice showing clinical signs of CCC support that TNF contributes to tissue damage, Cx43 loss, and electrical changes as arrhythmias, second-degree atrio-ventricular blockage (AVB-2), and prolongation of the PR and QTc intervals and QRS complex. In addition, TNF blockage reduced the frequencies of IL-17^+^CD4^+^ cells and TNF receptor 1 (TNFR1)-bearing CD8^+^ T cells, while increased the frequency of IL-10^+^ macrophages in spleen. Particularly, TNF blockage strategy placed TNF signaling as an upstream regulator of the balance of systemic proinflammatory (IFNγ, TNF and IL-17) and regulatory (IL-10) cytokines in the chronic infection. Crucially, TNF blockage downregulated TNF mRNA expression in the cardiac tissue and reduced infiltration by cytotoxic CD8^+^ T cells.^17^ In another study, administration of pentoxifylline (PTX), a phosphodiesterase inhibitor with hemorheological properties, to chronically *T. cruzi*-infected mice with signs of CCC reduced the frequency of TNFR1^+^CD8^+^ T cells. Moreover, PTX therapy restored the infection-induced TCR downregulation and improved parasite-specific CD8-mediated immune response. The repositioning of the abnormal CD8^+^ T cell response was associated with reversion of CCC traits, as electrical and echocardiographic abnormalities.^18^ Altogether, these data support a pivotal role for TNF/TNFR1 signaling in myocarditis formation, tissue injury, as well as conduction and functional changes in CCC.

In the acute phase, TNF and IFNγ are essential for chemokine-driven cell migration and cardiac tissue colonisation by inflammatory cells associated with parasite control. TNF and IFNγ also induce the production of reactive oxygen species (ROS) and other effector mechanisms crucial for control of parasite dissemination.^4^ ^,^ ^19^ However, in an apparent paradox, ROS fuels *T. cruzi* growth and is involved in functional impairment of the heart.^20^ More, ROS and oxidative stress induce deleterious effects on the respiratory chain of cardiac cell mitochondria in CD patients and *T. cruzi*-infected mice, resembling features of mitochondriopathies.^21^ ^,^ ^22^ ^,^ ^23^ In this context, reduction in the levels of the antioxidant enzymes glutathione peroxidase (GPx) and superoxide dismutase (SOD) were associated with the worsening of Chagas’ heart disease.^9^

In the chronic infection, the proinflammatory effector mechanisms (TNF, IFNγ, ROS) that contribute to parasite control may also cause tissue injury and clinical changes.^4^ ^,^ ^8^ ^,^ ^15^ ^,^ ^17^ ^,^ ^18^ ^,^ ^20^ Although regulatory effector mechanisms such as regulatory T cells, IL-10 and TGFβ may contribute to disease tolerance, lowering the microbicidal activity of macrophages in tissue, they also upregulate the production of arginase-induced putrescine, which fuels parasite growth.^24^ TGFβ also plays a direct role in parasite escape of the immune response, promoting cardiac cell invasion and parasite growth.^25^ Moreover, increased TGFβ plasma levels in the early chronic phase of CD were proposed as predictor of CCC progression and risk of death.^26^ In this context, CCC model in Colombian-infected C57BL/6 mice showed elevated TGFβ serum levels and TGFβ expression in heart tissue associated with increased expression and activity of the metaloproteinase MMP9, tissue remodeling and fibrosis. Crucially, blockage of TGFβ signaling using GW788388, a selective inhibitor of TβR1/ALK5, showed that this cytokine contributes to Cx43 loss and plays a fibrogenic role, worsening conduction and cardiac dysfunction.^27^ Altogether, these data support the complexity of cytokine contribution to CCC pathogenesis.

In CCC patients, myocarditis is composed of mononuclear cells, mainly cytotoxic CD8^+^(granzyme A^+^ cells), and CD4^+^ T cells and macrophages.^28^ In addition, elevated serum levels of CC-chemokine ligands and CC-chemokine receptors on T-cells and macrophages are related with severity of Chagas’ heart disease.^8^ ^,^ ^23^ Thus, a role for CC-chemokines in the coordination of migration of these cells towards the cardiac tissue has been proposed. In the acute and chronic infection of C3H/He and C57BL/6 mice with the Colombian strain, CC-chemokine concentrations in the heart tissue are related with the intensity of inflammation.^29^ ^,^ ^30^ Studies using mouse lineages deficient in CC-chemokine ligand (CCL3) or receptor (CCR5) and selective CCR1/CCR5 antagonist revealed that CCL3, CCL4 and CCL5 ligands, acting via the CCR1/CCR5 receptors, control the migration of T cells and macrophages towards the cardiac tissue, cause cardiomyocyte lesion and conduction and functional abnormalities.^29^ ^,^ ^30^ ^,^ ^31^ ^,^ ^32^ Moreover, CCL3 was posed as a hub in CCC pathogenesis, regulating the intensity of TNF- and IFNγ-enriched inflammatory milieu, the degree of cardiomyocyte injury, prolonged QTc interval and cardiac dysfunction.^30^ Hence, these findings reinforce a role for TNF and IFNγ in *T. cruzi*- triggered cardiomyocyte damage, and conduction and functional changes, pivotal features of CCC.

The molecular mechanisms that underpin the pathogenesis of CCC to be unraveled are urgent need. In the cardiac tissue of patients with CD and in CCC model using Colombian-infected C57BL/6 mice,^33^ ^,^ ^34^ ^,^ ^35^ upregulation and downmodulation of the expression of certain microRNAs (miRNAs), molecules that specifically control mRNA translation, have been described. In acutely Colombian-infected C57BL/6 mice, predictive analysis supports the correlation of changes in miRNAs (miR-21, miR-145-5p and miR-146b-5p) in the cardiac tissue with parasitaemia levels and prolonged QTc interval, and with molecules essential for cardiac activity, as metabolite and ion channels (as GJA5, KCNA1). Further, target gene network analysis supports the association of pivotal miRNAs with the expression of relevant molecules, biological pathways, signaling signatures and mitochondrial dysfunction, placing IFNγ, TNF, NFκB as upstream regulators of miRNAs and mRNAs.^35^ ^,^ ^36^ Crucially, these findings were recently supported by analysis of the heart of CCC patients.^34^ Also, miR-155 miRNA has been associated with *T. cruzi* infection control and serum levels of the inflammatory cytokines IFNγ and TNF.^37^ Moreover, a recent study showed that co-exposure of human cardiomyocytes to IFNγ and TNF induces nitro-oxidative stress and mitochondrial dysfunction,^38^ reinforcing the direct effect of these proinflammatory cytokines in cardiac cell injury.

Increased TNF and soluble TFNR1 (sTNFR1) serum levels, biomarkers of systemic inflammatory diseases, are detected in chronic CD patients regardless of clinical forms.^39^ Severity of CCC is correlated with high serum levels of proinflammatory (IL-6, IL-17, IFNγ, TNF) and regulatory (IL-4, IL-10, TGFβ) cytokines, chemokines (CCL2, CCL3, CCL5), and the inflammatory mediators NO.^9^ ^,^ ^10^ ^,^ ^11^ ^,^ ^40^ Although most evidence lacks causal correlation, these data suggest that CCC is associated with a systemic inflammatory profile. Regardless the academic requirements for causa-effect relation in CCC patients, in experimental models subjected to therapeutic interventions with an adenovirus-based vaccine,^15^ anti-TNF,^17^ PTX,^18^ TGFβ signaling blocker^27^ or the trypanocidal drug Bz^41^ the results support that lowering the systemic inflammatory cytokine levels plays beneficial role in CCC progression. Reinforcing this idea, in a study performed in an indeterminate model of CD, low serum cytokine levels, like those of uninfected controls, parallel low-grade cardiac parasitism and inflammation in the absence of clinical signs of heart disease.^42^

Here, we bring a cartoon summarising data obtained in CD patients and preclinical studies and a proposal to Chagas’ heart disease pathogenesis. In the acute phase of infection, low inflammatory cytokines and NO levels are crucial for parasite control. However, as infection progresses to chronic phase, low-grade parasitism persists, and TNF, sTNFR1 and NO serum levels are associated with disease severity. Tissue infiltration by inflammatory cells is sustained and this long-lasting scenario may contribute to tissue damage with CC-chemokines / inflammation, TNF-and IFNγ-enriched milieu, miRNA/mRNA dysregulation, ion channels changes, ROS increase, oxidative stress, mitochondrial dysfunction, increased MMP9 activity, hypertrophy, tissue remodeling and fibrosis, thus contributing to conduction and functional changes (Fig. 1). Altogether, the above discussed studies highlight the pivotal role of signaling pathways triggered by proinflammatory cytokines (particularly, IFNγ and TNF) in heart tissue injury, as well as conduction and functional activities, opening perspectives for the comprehension of the molecular mechanisms underlying the pathogenesis of CCC and the proposal of trait-based immunomodulatory strategies.

**Fig. 1:**
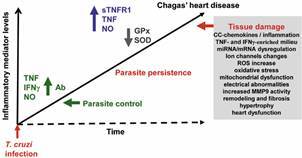
cytokines and inflammatory mediators may take part in parasite control but may also play a role in the pathogenesis of Chagas’ heart disease, fueling inflammation-related tissue damage. In the acute phase of the infection, antibodies (Ab) and low levels of cytokines (as TNF, IFNγ) and inflammatory mediator (as NO) are crucial for parasite control. As infection progresses to chronic phase, TNF, sTNFR1 and NO serum levels are associated with chronic chagasic cardiomyopathy (CCC) severity. Parasite persistence and this long-lasting systemic inflammatory profile scenario may contribute to inflammation-related heart tissue damage with increased expression of CC-chemokines / inflammation, TNF-and IFNγ-enriched milieu, miRNA/mRNA dysregulation, changes of ion channels expression, reactive oxygen species (ROS) increase, oxidative stress, mitochondrial dysfunction, increased MMP9 activity, hypertrophy, tissue remodeling and fibrosis, thus contributing to conduction and functional changes.

Multi-therapeutic strategy targeting parasite and inflammation-related alterations impacts CCC progression

A bunch of evidence support multifactorial contributions to CCC as (i) parasite persistence, (ii) inflammation-related tissue damage, and (iii) systemic inflammatory profile. Keeping this in mind, we carried out a hypothesis-based study testing effectiveness of a reduced dose of Bz (25 mg/Kg/day, 1/4 of the usual dose, 30 consecutive days), aiming at diminishing parasite load, combined with PTX, to use immunoregulatory properties to interfere with traits associated with CCC.^18^ Our idea was challenged in chronically Colombian-infected C57BL/6 mice showing clinical signs of CCC. Several analyses were carried out, as shown in our previous publications.^41^ ^,^ ^43^ Briefly, low dose of Bz was effective in heart parasite control with no significant impact on electrical abnormalities, at the end of treatment. However, 30 days after treatment cessation conduction changes were improved,^41^ suggesting that time is required to recover tissue damage. Indeed, in a long-term follow-up, Bz therapy was associated with decreased incidence of CD progression form indeterminate to cardiac form and cardiac events.^44^ In our study, PTX did not interfere with Bz-induced parasite control but presented partial beneficial effects on conduction alterations. Moreover, after Bz+PTX therapy conduction changes were improved.^41^ Particularly, the QTc prolongation, a biomarker associated with cytokine-related inflamed heart tissue,^45^ seen in vehicle-treated (Veh) infected mice was restored, alike uninfected controls, in Bz+PTX-treated mice (Fig. 2A). Importantly, the three therapeutic schemes reduced heart fibrosis.^43^ Moreover, Bz therapy reduced the levels of TNF mRNA expression in heart tissue and sTNFR1 serum levels.^41^ Therapy with suboptimal dose of Bz mitigated Th1-driven molecular pathways detected in Veh-treated group, while combined Bz+PTX modulated cell death and survival pathways, which may improve CCC progression.^43^ Crucially, Bz and Bz+PTX strategies downregulated IFNγ, an upstream regulator of miRNA and mRNA expression in the heart tissue.^43^ Here, a new glance was lanced at these data,^43^ focusing on a group of molecules that may contribute to inflammation-related alterations playing a role in major biological processes as cell migration (CCL3, CCL5, CXCL10, CXCL11, CXCR3), myocarditis formation (CD3ε, CD8a, CD4, CD19), inflammation (IFNγ, IL-10, IL-12a, IL-15, IL2R, IL-1Ra, IL-6, IL-7), and cytotoxic activity of T-cells (CTL; granzyme, perforin1). In comparison with uninfected controls, Veh-treated infected mice present a global increase in the expression of these molecules in the heart tissue, reduced by suboptimal dose of Bz and, mainly, by the combined Bz+PTX therapy (Fig. 2B). Therefore, rational treatment with suboptimal dose of the trypanocidal drug Bz reduces parasitism and combined with the immunoregulator PTX shows modulatory effects on a group of relevant upregulated genes in experimental CCC. This new scenario may reduce the continuous cell migration towards heart tissue and inflammation-related changes, which may allow tissue healing and improve disease tolerance. Thus, multi-therapeutic strategy targeting parasite and inflammation-related alterations may add beneficial effects to improve CCC prognosis and, even, reverse chronic cardiac alterations.

**Fig. 2:**
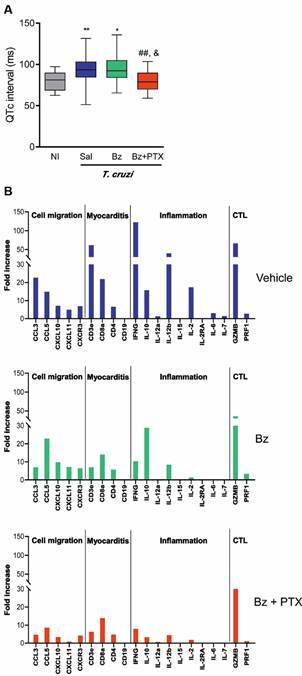
multi-therapeutic strategy targeting parasite and inflammation-related tissue changes impacted experimental chronic chagasic cardiomyopathy (CCC). Colombian-infected C57BL/6 mice showing clinical signs of CCC received vehicle (Veh), suboptimal dose of the trypanocidal drug benznidazole (Bz) or Bz plus the immunomodulator pentoxifylline (Bz+PTX). (A) The prolonged QTc, seen in vehicle-treated (Veh) infected mice was not impacted by Bz therapy, but restored, alike uninfected controls, after Bz+PTX therapy.^41^ (B) Data on gene expression in the heart tissue were reanalysed,^43^ focusing on the expression of molecules associated with cell migration (CCL3, CCL5, CXCL10, CXCL11, CXCR3), myocarditis (CD3ε, CD8a, CD4, CD19), inflammation (IFNγ, IL-10, IL-12a, IL-15, IL2R, IL-1Ra, IL-6, IL-7), and cytotoxic activity of T-cells (CTL; granzyme, perforin1). Compared with uninfected controls, in Veh-treated infected mice most of these genes were upregulated. Therapy with Bz and, crucially, Bz+PTX, mitigated the expression of these genes.

Final remarks

Besides our results discussed above, Fig. 3 summarises potential pharmacological and non-pharmacological candidate strategies to confront the complexity of the molecular mechanisms underpinning the pathogenesis of Chagas’ heart disease and improve prognosis, regarding the three major factors that may sustain CCC: (i) parasite persistence, (ii) intrinsic cardiac alterations, and (iii) immunological unbalance in heart and systemic inflammatory profile. To control parasite persistence, patients may benefit of protocols lowering doses of Bz or using short-term administration schemes, to preclude side effects. Certainly, novel drugs targeting parasite biological circuits may offer new opportunities to eliminate infection,^6^ ^,^ ^46^ and even present the possibility of a drug cocktail targeting parasite for CD therapy. Considering intrinsic cardiac alteration, Chagas’ heart disease patients may benefit of pharmacological therapies currently used to treat other cardiac conditions. Some signs- and symptom-based pharmacological therapies are indicated to treat CD patients, as the antiarrhythmic agent amiodarone and drugs to interfere with the dysfunctional neurohormone circuits, as renin-angiotensin-aldosterone system (RAAS), inhibitors of angiotensin converting enzyme (ACEi) and angiotensin receptor blockers (ARB).^1^ ^,^ ^3^ Recent findings also suggest that antioxidant therapies, also considered as nutritional approaches, as resveratrol and selenium, may be advantageous to CCC prognosis.^47^ ^,^ ^48^ ^,^ ^49^ Immunoregulators as PTX, safely used in other cardiopathies, and immunological and hematological conditions,^50^ may be an alternative to reeducate the immune system and control immunological unbalance in the heart tissue and systemic inflammatory profile.^18^ ^,^ ^41^ ^,^ ^43^ Obviously, we should further explore other immunomodulators to ameliorate CCC, as reeducation of the immune system by therapeutic vaccines,^15^ and immunoregulatory properties of other drugs as Bz, which reduces TNF expression and NF-κB signaling.^51^ Lastly, non-pharmacological strategies as physical exercises may contribute to improve CCC prognosis.^52^ One should keep in mind that further studies are required to unveil the molecular mechanisms underpinning CCC and to add new alternatives to this quite short list. Thus, the available knowledge reasonably supports the proposal of multi-therapeutic schemes to be challenged in clinical trials to confront the complexity of CD.

**Fig. 3:**
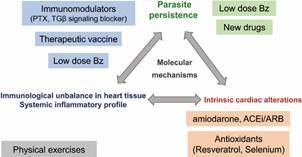
pharmacological and non-pharmacological potential candidates to compose multi-therapeutic strategies to confront the complexity of Chagas’ heart disease. Three major factors may sustain chronic chagasic cardiomyopathy (CCC): (i) parasite persistence (target by Low doses of Bz; New drugs), (ii) intrinsic cardiac alterations (target by the antiarrhythmic agent amiodarone; inhibitors of angiotensin converting enzyme - ACEi - and angiotensin receptor blockers - ARB; antioxidant agents or nutritional approaches using resveratrol and selenium), and (iii) immunological unbalance in heart and systemic inflammatory profile (target by immunoregulators as PTX and TGF signaling blocker, therapeutic vaccines, immunomodulatory properties of Low dose of Bz). Non-pharmacological strategies as physical exercises may also contribute to improve CCC prognosis.

One and all should be aware that crucial points shall be overcome to challenge a multi-therapeutic strategy in Chagas’ heart disease: (i) the bottle neck access of CD patients to screening and clinical follow-up; (ii) the deceptive pursuit for the silver bullet for the etiological treatment of CD patients; (iii) the lack of recognition that CD is an multi-factorial disease, with a parasite-triggered systemic inflammatory profile and other pivotal biological pathways; (iv) the lack of consensual biomarkers to follow-up effects of therapeutic protocols; and (v) the valley of death separating preclinical and clinical trials to treat neglected tropical diseases, as CD. Among the new perspectives in a post-pandemic world, all the recent advances in the comprehension of the host-invader interplay, drug and vaccine development, may allow more receptive minds to test vaccines and new therapeutic strategies accessible to millions of people afflicted by neglected diseases, as Chagas disease.
